# Efficient Drug Screening and Nephrotoxicity Assessment on Co-culture Microfluidic Kidney Chip

**DOI:** 10.1038/s41598-020-63096-3

**Published:** 2020-04-16

**Authors:** Lei Yin, Guanru Du, Bing Zhang, Hongbo Zhang, Ruixue Yin, Wenjun Zhang, Shih-Mo Yang

**Affiliations:** 1 School of Mechanical and Power Engineering, East China University of Science and Technology, Shanghai, P.R. China; 2 Biomedical Science and Technology Research Center, School of Mechatronic Engineering and Automation, Shanghai University, Shanghai, P.R. China; 3 Division of Biomedical Engineering, University of Saskatchewan, Saskatoon, Canada

**Keywords:** Lab-on-a-chip, High-throughput screening

## Abstract

The function and susceptibility of various drugs are tested with renal proximal tubular epithelial cells; yet, replicating the morphology and kidneys function using the currently available *in vitro* models remains difficult. To overcome this difficulty, in the study presented in this paper, a device and a three-layer microfluidic chip were developed, which provides a simulated environment for kidney organs. This device includes two parts: (1) microfluidic drug concentration gradient generator and (2) a flow-temperature controlled platform for culturing of kidney cells. In chip study, renal proximal tubular epithelial cells (RPTECs) and peritubular capillary endothelial cells (PCECs) were screened with the drugs to assess the drug-induced nephrotoxicity. Unlike cells cultured in petri dishes, cells cultured in the microfluidic device exhibited higher performance in terms of both cell growth and drug nephrotoxicity evaluation. It is worth mentioning that a significant decrease in cisplatin-induced nephrotoxicity was found because of the intervention of cimetidine in the microfluidic device. In conclusion, the different in the cell performance between the microfluidic device and the petri dishes demonstrates the physiological relevance of the nephrotoxicity screening technology along with the microfluidic device developed in this study. Furthermore, this technology can also facilitate the development of reliable kidney drugs and serve as a useful and efficient test-bed for further investigation of the drug nephrotoxicity evaluation.

## Introduction

Developing and commercializing new drugs is a long and costly process. Pharmaceutical companies typically spend a few decades and substantial amounts of money for drug research and development (R&D)^1^. However, due to nephrotoxicity, 2% and 19% of the new drugs fail in preclinical trials and phase III clinical trials, respectively^2^. The current approach for evaluating drug nephrotoxicity is based on cell models and animal experiments^3,4^; yet, they cannot provide high-fidelity outcomes. Moreover, there are many controversies regarding funding and ethics in animal experiments. It is therefore highly desirable to predict drug-induced nephrotoxicity without a need of animal experiments. This could be achieved using intelligent devices called organ-on-chip. Today, the organ-on-chip technology is very promising in drug screening^5–7^. Kidney plays the functions of generating urine and retaining nutrients from re-absorption^8^. To maintain homeostasis, water channels and renal transporters are localized on the upper and basolateral membranes of renal tubular epithelial cells^9–11^.

Nephron, consisting of the glomerulus, renal capsule, and renal tubule, is the main component in kidney^12^. The renal function is evaluated by analyzing urea nitrogen and serum creatinine. However, this evaluation approach often lacks accuracy and sensitivity^13,14^. Further, due to changes in the experimental method as well as patients’ cases, detection of these indexes cannot reflect early kidney damage. Therefore, there is an urgent need to develop an early predictive cell model for the *in vitro* drug nephrotoxicity evaluation, which has a significant benefit to new drugs development.

Recently, the reconstruction of an environment with renal tubular cells has attracted considerable attention in the field of microfluidics, as such an environment has a potential to imitate a real cell environment. In such an environment, the fluid flows and interacts with the kidney tissue or cell to create shear stress in the tissue or cell^15,16^. It has been shown that shear stress could cause junctional reformation and cytoskeletal reorganization for renal proximal tubular epithelial cells^17,18^. Further, shear stress also alters the expression of apical and basolateral transporters and regulates sodium ions^19,20^. Finally, when exposed to the fluidic condition, the kidney epithelium to the interstitial space may show an active transport of glucose and amino acids across the epithelium^21^.

Microfluidics provides an excellent tool to develop the aforementioned artificial cell environment^22–24^. Currently, such environments have been applied to several artificial organs such as the lung, liver, kidney, and gut^25,26^. A salient point concluded from these works is that such environments must be of 3D structures^27–31^ for applications including drug screening^32,33^ for organs including kidney^34^. As such, in our previous work^35^, we developed a kidney environment by means of microfluidics technology for renal tubules and perivascular capillary culture. That environment system included a concentration gradient chip and a flow-temperature control device for long-term cell culture. The study reported in the present paper was a continuous effort to explore the feasibility of using that system for predicting drug-induced nephrotoxicity via cisplatin (DDP), gentamycin (GM), and cyclosporin A (CsA) on the kidney chip.

This article observes the permeability effect of the membrane on the chip. In terms of different concentrations of reagents, concentration gradient chip was used to generate 5 kinds of drug concentrations, and increase the rate of concentration. In terms of drug detection, the effect of GM on cells for only 1 day was previously measured on the chip. This article measures the effects of DDP, GM, and CsA on cells at 1, 4, and 7 days. More importantly, we also used Cim to build an attenuation model. The results of these new studies expected to build a reliable artificial kidney disease model to predict effects of drugs on human body and to complete the establishment of drug detoxification model.

## Materials and methods

### Principle of kidney system

**Figure 1:**
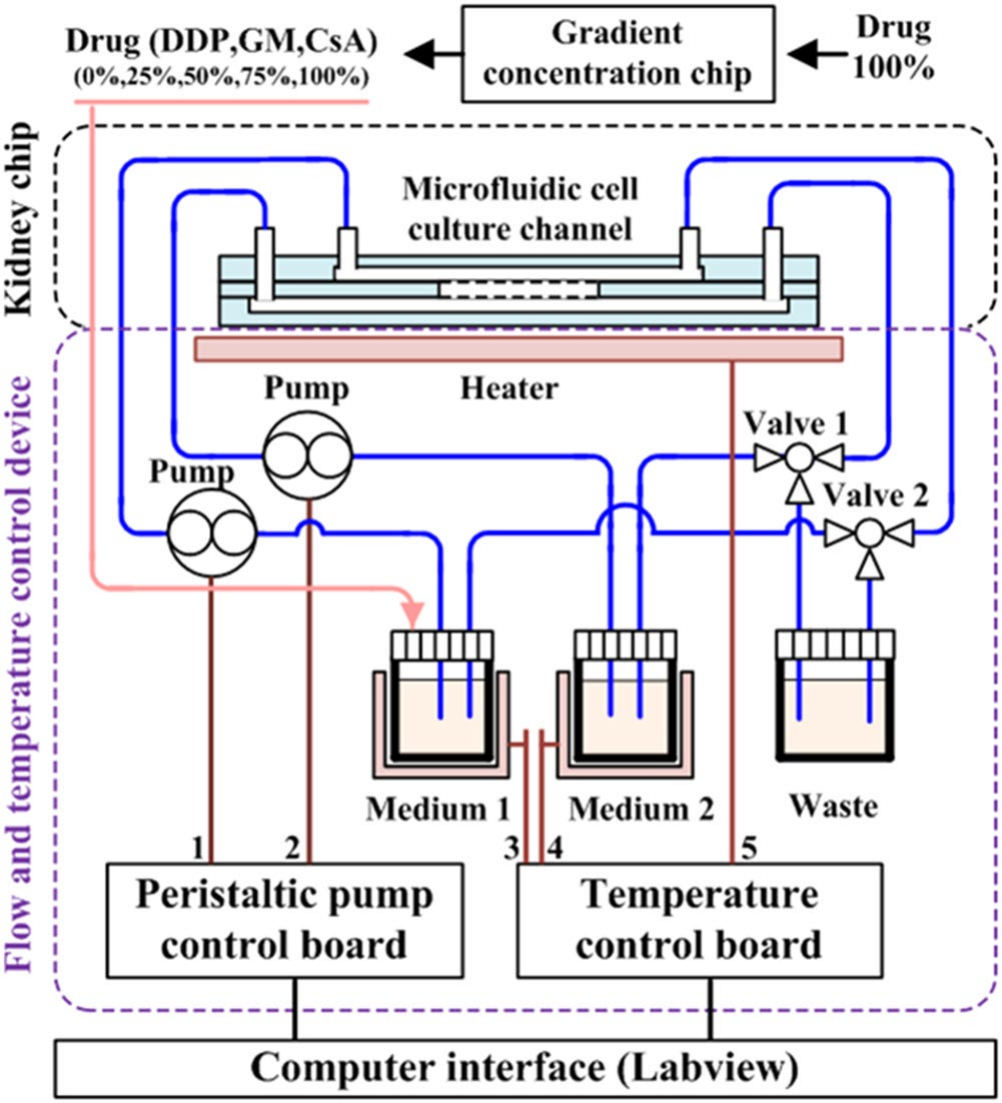
By adjusting the heating plate, the temperature of the chip and the cell culture medium can be maintained. Two pumps can regulate the flow rate of the cell culture medium into the chip.

In general, an organ-on-chip system includes three parts: drug concentration gradient generator, three-layer microfluidic organ chip, and flow-temperature control device. In the study reported in this paper, the kidney organ was concerned. In the study, first, two groups of drug concentrations were performed on the gradient chip. Second, RPTECs and PCECs were cultured individually on opposite membranes. After treatment with a drug, RPTECs’ viability was quantified to determine the effect of that drug. Third, a flow-temperature control device was operated to stabilize the conditions for long-term cell culture. This device mainly comprised in three parts (Fig. 1): two miniature peristaltic pumps, a temperature control device, and matching catheters.

**Figure 2:**
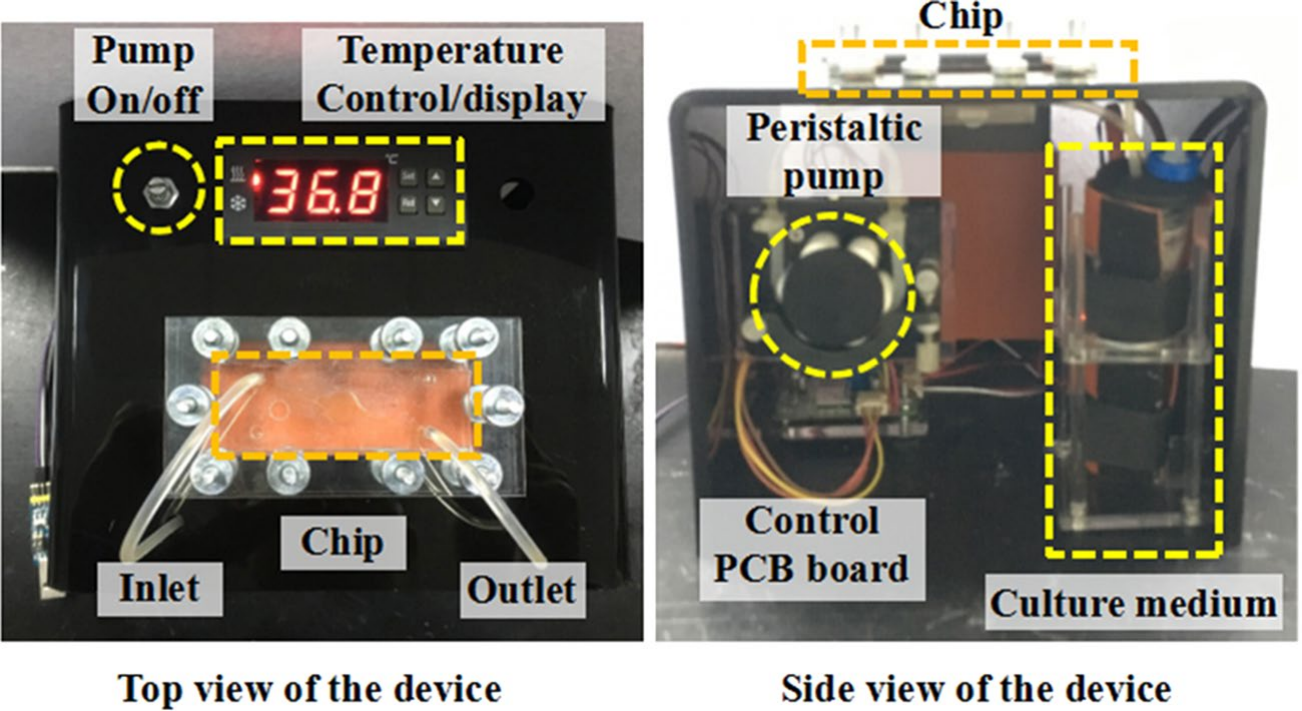
Hardware diagram of the culture platform that provides an automated method to control the temperature and medium flow for long-term cell culture.

The peristaltic pump controls the high-precision flow rate within the range of 10~100 μl/min. The temperature control comprises a thermostat (AT8017 microcomputer temperature controller), heating plate (unit power: 0.5 W/cm^2^), and temperature sensor. The heater controlled two parts. First, it was attached to the bottom of the microfluidic kidney chip to maintain its operating temperature. Second, it maintained the circulation temperature for the culture medium stored in the tube. Silicone rubber was selected as the heating material, because it has a high heating speed, strong plasticity, and a good heat resistance of physical and chemical properties. In this experiment, it took almost seven days for the cells to grow into a layer. If the experimenter chooses other cells that grow faster, the time to grow into a layer will be shortened. In the design of the experiment, what we call the long-term cultivation is that we can cultivate for 7 days. Within this time, the required data can be obtained. Figure 2 shows the top and side views of the device.

### Chip fabrication

#### The three-layer microfluidic kidney chip

**Figure 3:**
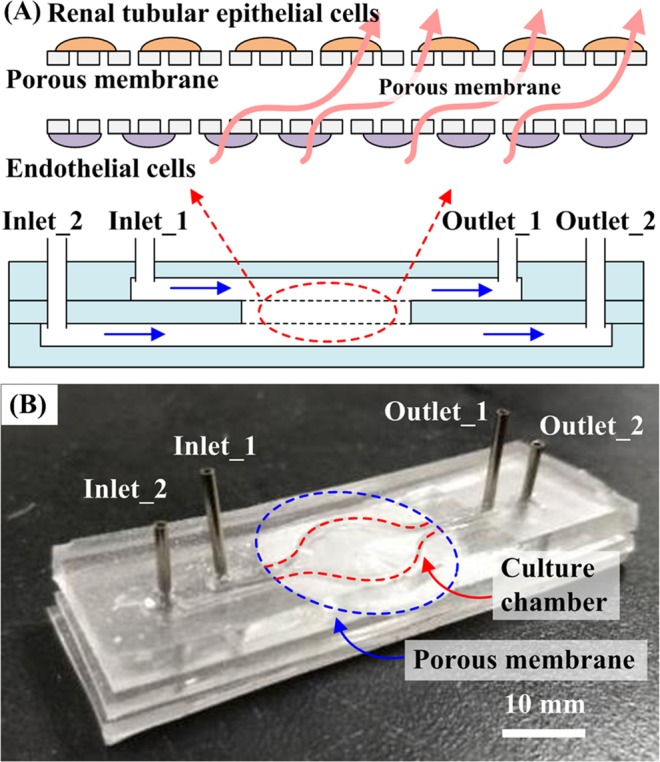
Design of the three-layer microfluidic kidney chip. (**A**) Schematic of the chip. RPTECs were cultured in the presence of a physiological level of apical fluid shear stress. PCECs were cultured at the back of the membrane. (**B**) The microfluidic kidney chip comprises two inlets, two outlets, two membranes, and three PDMS layers.

This chip validates the feasibility of growing the two cells on opposing membranes. AutoCAD 2016 software was used to define the channel pattern (width: 0.5 mm, height: 1 mm, cell culture diameter: 14 mm) and to produce the mold by laser-cutting poly (methyl methacrylate) (PMMA). The PDMS prepolymer mixed solution [Sylgard 184 A and B (10:1 w/w)] was cast on the mold and placed on a hotplate with T = 70 °C for 2 h. After curing by 30 mins, the PDMS layers were peeled-off, and two inlet and two outlet holes were cut and punched. The length of tubing was within 300 mm. Two polyester membranes (pore size: 0.4 µm, thickness: 10 µm, diameter: 16 mm) were sandwiched between two PDMS layers, and the whole assembly separated each of the chambers, Fig. 3(B). The PDMS surface was treated with O_2_ plasma for bonding. The secretion of both cells can be exchanged through the membrane, Fig. 3(A).

The RPTECs and PCECs adhered the upper and bottom layers of the chip adhered. The cells were exposed to a fluid shear stress of 0.2 dyne/cm^2^. The flow rate can be calculated using the equation: σ = 6uQ/H^2^W, where u is the medium viscosity at 37 °C (gm/cm/s); Q, is the volumetric flow rate (cm^3^/s); W is the channel width; and H is the channel height^19^. (σ = 0.2 dyne/cm^2^, u = 0.7×10^−3^ pa.s, H = 0.5 mm, W = 1 mm.)

It is well known that micro-channels in tiny spaces are easy to trap bubbles. To prevent this phenomenon from happening, we added 10% alcohol liquid into the chamber. It can quickly drive bubbles out of the gap. After 30 seconds, the PBS washed the channel for 10 minutes to ensure that there is no alcohol in the chip. To prevent the cross infection between different drug concentrations, the chip is disposable.

It must be emphasized that our co-culture system is not to mix the RTPECs and PCECs and put them into a chip. These two cells are grown on different membranes. Therefore, we will not see the two cells in the same picture.

#### The concentration gradient chip

**Figure 4:**
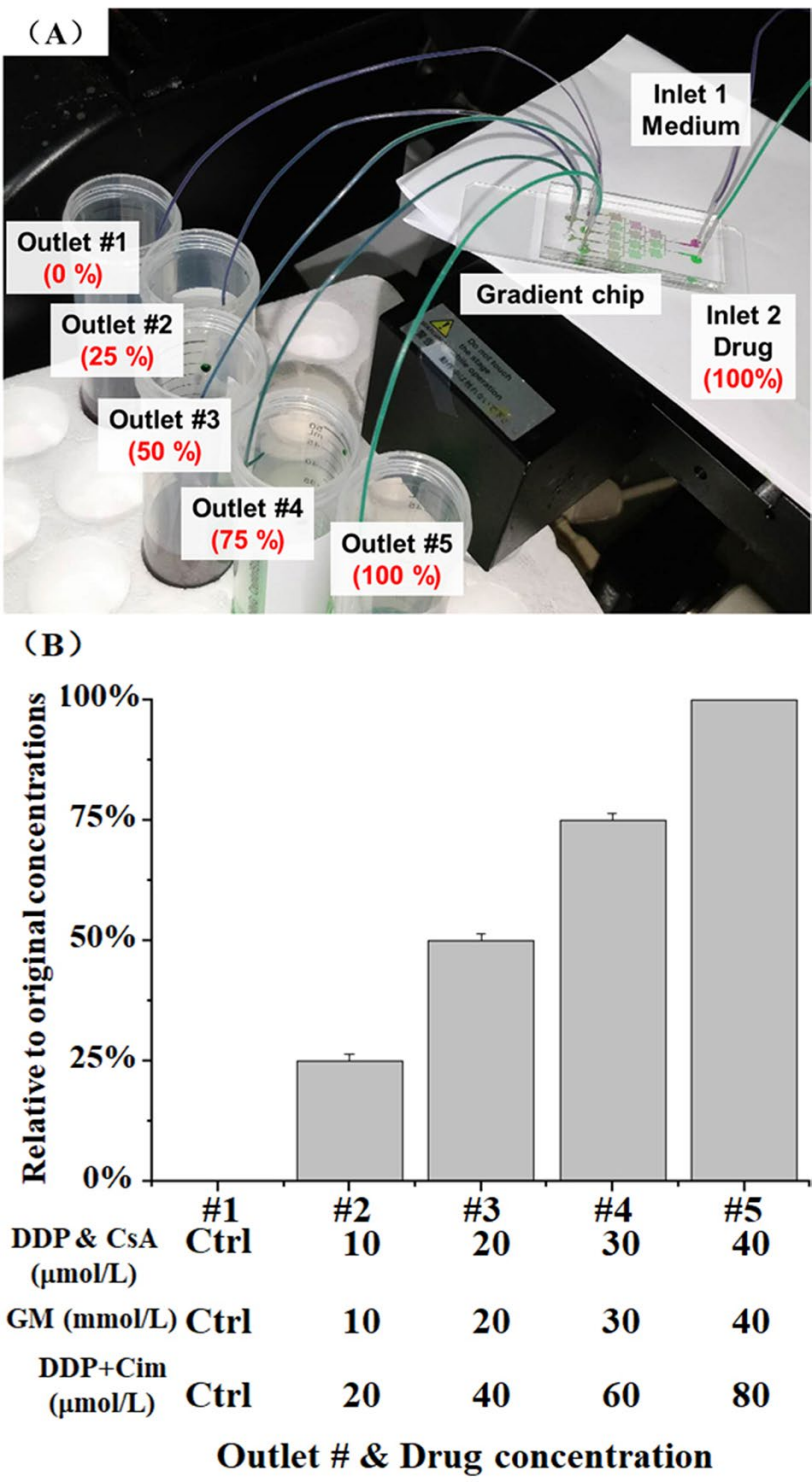
Schematic graph of CGC. (**A**) Drugs (DDP, GM, and CsA) were injected and mixed with the culture medium within the gradient network. Five different uniform concentrations (0%, 25%, 50%, 75%, and 100%) were collected at the outlets. (**B**) Gradient concentration distribution at different outlets. Different drug concentrations of DDP, GM, and CsA could be collected via this chip. The data are presented as mean ± S.D.

The channel on the concentration gradient chip (CGC) was designed as a Christmas tree; see Fig. 4 (A). We firstly used red and green food colorants to get the best operating conditions for 0% (control), 25%, 50%, 75%, and 100% concentration. The concentration distribution can be quantified by analyzing the RGB value of the solution color; see Fig. 4 (B). Next, the same parameters of flow rate, 10~100 μl/min, were applied to obtain different drug concentrations. In particular, we pumped a medium containing 40 μmol/L of DDP from inlet_2 and no-drug medium from inlet_1, five different concentrations, 0, 20, 20, 30, and 40 μmol/L, were collected from outlets 1–5, respectively.

### Membrane permeability

**Figure 5:**
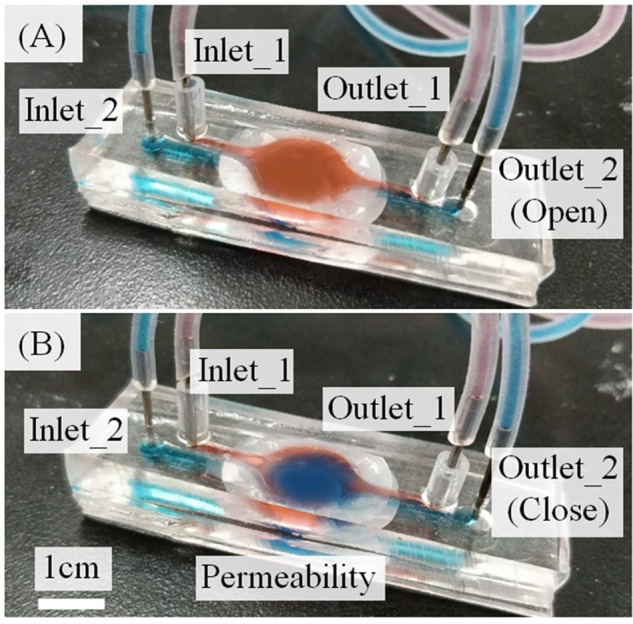
Permeability test of the microfluidic kidney chip. (**A**) First, the upper layer was red in color. (**B**) After perfusion, the upper layer gradually showed blue color. The culture medium passes through the membranes from the lower to the upper channel.

We used food colors to test the permeability of the membrane. The red and blue pigments in the upper and lower layers were perfused constantly (Fig. 5). As outlet_2 was closed, the blue medium in the lower microchannel permeates through the membrane into the upper layers. The pigment of the upper layer gradually became lighter and a blue part appeared after 5 minutes. We used this operation to achieve the infiltration of kidney tissue.

### The cell culture process

Renal proximal tubule epithelial cells (RPTECs) have the complete transporter and metabolic enzyme expression; polarized tight monolayer formation; trans-epithelial transport. Peritubular capillary endothelial cells (PCECs) are also the representative human capillary cells. RPTECs and PCECs were selected for this experiment and cultured in high-glucose Dulbecco’s modified Eagle’s medium (DMEM) with a density of 5 × 10^5^/mL.

First, the polycarbonate membranes were coated with extracellular matrix (ECM) collagen to enable the cell adhered to the membrane surface. The density of cells in the chip is controlled by adjusting the concentration and volume of the injected cells, for which the cell concentration of 5 × 10^4^/mL is enough. Second, PCECs were loaded into the lower channel and cultured for 4 h. As PCECs adhered to the membrane, the microfluidic kidney chip was placed upside down, and RPTECs were loaded into the upper channel. As the cell rested statically for 4 h, the microfluidic kidney chip was placed on the flow-temperature control device for long-term culture. Finally, the entire system, chip, and device were placed in a 5% CO_2_ incubator.

In this experiment, it took almost seven days for the cells to grow into a layer. If the experimenter chooses other cells that grow faster, the time to grow into a layer will be shortened. In the design of the experiment, what we call the long-term cultivation is that we can cultivate for 7 days. Within this time, the required data can be obtained.

### Immunofluorescence staining

 Fluorescence images of live cells (green, Calcein-AM) and dead cells (red, propidium iodide) were analyzed by CCK-8 assay in the time points: 1 day, 4 days, and 7 days. RPTECs were treated with three conditions: culture on a petri dish, culture in the chip, and co-culture with PCECs in the chip. To the first and second conditions, the cells were cultured for the time durations as mentioned before, and then, the CCK-8 solution (CCK-8 reagent: culture medium = 1:10) was added to the membrane 1 and incubated for 2 h. Immediately after that, the cell metabolism products were collected from outlet_1 and outlet_2. In the co-culture system, it was found that the culture medium collected from the outlets contained the metabolite from both RPTECs and PCECs. Therefore, the membrane with RPTECs was taken and cultured in the dish for 2 hours for CCK-8 assay. This step prevented the influence from PCECs. After that, the CCK-8 solution was aspirated into a 96-well plate (120–150 µL per well), and measured with 450 nm wavelength. It is worth to mention that the study reported in this paper was focused on the drug assay of RPETCs, which explains our steps towards all the results related to RPETCs.

### Drug nephrotoxicity screening

 DDP, GM, and CsA were chosen for nephrotoxic drugs. First, DDP is a typical recognized nephrotoxic drug for the clinical application and usually used for lung and esophageal cancer, soft neoplasms, and other solid tumors. Cisplatin-induced nephrotoxicity mechanisms include the following: DDP can make renal blood vessels shrink gradually, impacting the glomerular filtration rate and resulting in renal damage, proteinuria, and other symptoms^36^. Second, GM is another clinical aminoglycoside in the antibiotic category and mainly treats diseases induced by gram-negative bacteria, *Neisseria gonorrhoeae*, and other infections. We chose GM as a test drug due to its strong nephrotoxicity^37,38^. Third, CsA is mainly used for dealing with the rejection of organ transplants such as the heart, liver, and kidney. The nephrotoxicity of CsA makes it be appropriate for the disease model test^39,40^.

Two groups of DDP concentration (0, 20, 40, 60, and 80 μm/L and 0, 20, 40, 60, and 80 μm/L) and Cim (1 mm/L) were injected into the top chamber to verify the function of Cim for reducing DDP-induced toxicity. The doses were picked from the literature^41,42^. We compared the drug-induced nephrotoxicity of the Petri dishes (static) and microfluidic kidney chips (fluidic) by cell morphology, live/dead staining, and CCK-8 assay. Cells were divided into three groups based on their gradient concentrations:

1. DDP group: 0, 10, 20, 30, and 40 μmol/L;
2. GM group: 0, 10, 20, 30, and 40 mmol/L;
3. CsA group: 0, 10, 20, 30, and 40 μmol/L.

### Materials

The chips were made from the following materials: PDMS (Dow Corning, Midland, MI, USA) and polyester porous membrane (pore size: 0.4 mm; Whatman, Maidstone, UK). The following materials were used for the cell culture: DMEM, fetal bovine serum (FBS), penicillin-streptomycin, phosphate-buffered saline (GIBCO BRL, Grand Island, NY); dimethyl sulfoxide (Sigma, St.Louis, MO); Collagenase B (Roche, Mannheim, Germany); and DDP, GM, CsA, and Cim (Sigma). CCK-8 was purchased from Dongren Chemical Technology Co., Ltd. (Shanghai). The live/dead and DAPI reagents were obtained from Shanghai Enyi Biological Technology Co., Ltd. RPTECs and PCECs were purchased from Shanghai Saige Biological Co., Ltd.

## Results and discussion

### RPTECs viability analysis

**Figure 6:**
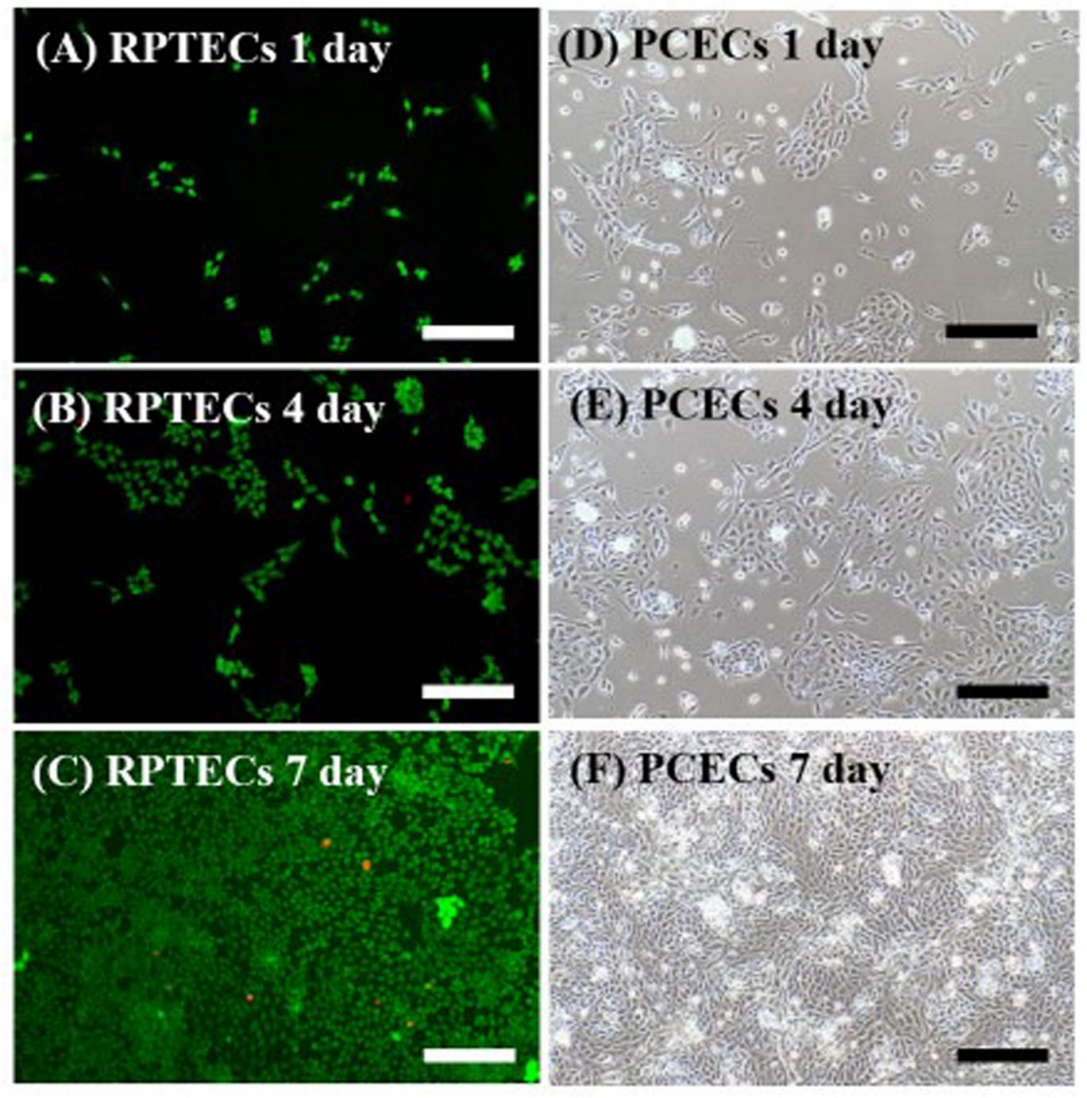
(**A**–**C**) are the RPTECs results of the chip culture on 1, 4, 7 days. Live and dead cells were labeled in green and red, respectively. (**D**)–(**F**) is the PECEs results on 1, 4, 7 days. Scale bar = 200 μm.

The morphology of cells in the chip was recorded and analyzed by ImageJ software (Fig. 6). The cell viability of RPTECs on-chip was 89.6% and 90.2% on the 4^th^ and 7^th^ days by CCK-8 assay. It is higher than that on the Petri dishes, 87.1% and, 88.5%. For the co-culture system, the viabilities were 91.3% and 92.7%.

**Figure 7:**
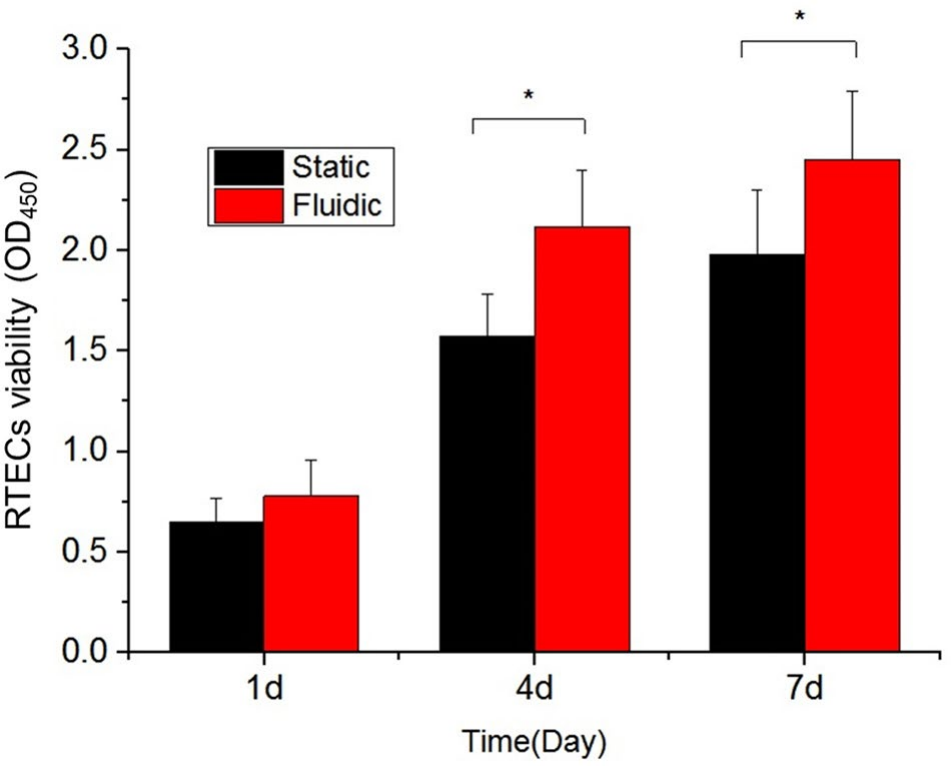
CCK-8 assay of RPTECs under static, fluidic, and fluidic co-culture conditions on day 1, day 4, and day 7. The fluidic environment with flesh culture medium circulation provides more favorable culture conditions for cell growth. The OD value represents the cell viability.

Figure 7 shows the analysis of three cell culture conditions, on the Petri dish, the microfluidic chip, and the co-culture system. Cell culture conditions, such as the density of the loaded cells, are the same as in Fig. 6. CCK-8 indicates the number of cells in terms of OD value; the higher the OD value, the larger is the number of viable cells (Fig. 7). The percentage of live cell was higher in the fluidic condition of the microfluidic chip than in the static group on the 4^th^ and 7^th^ days (p < 0.05). The RPTECs had higher viability as PCECs cultured on opposite membranes. This means the microfluidic-based co-culture kidney chip model is a suitable platform for long-term cell culture and for testing the drug function effect for drug screening.

### Drug test the on microfluidic kidney chip

**Figure 8:**
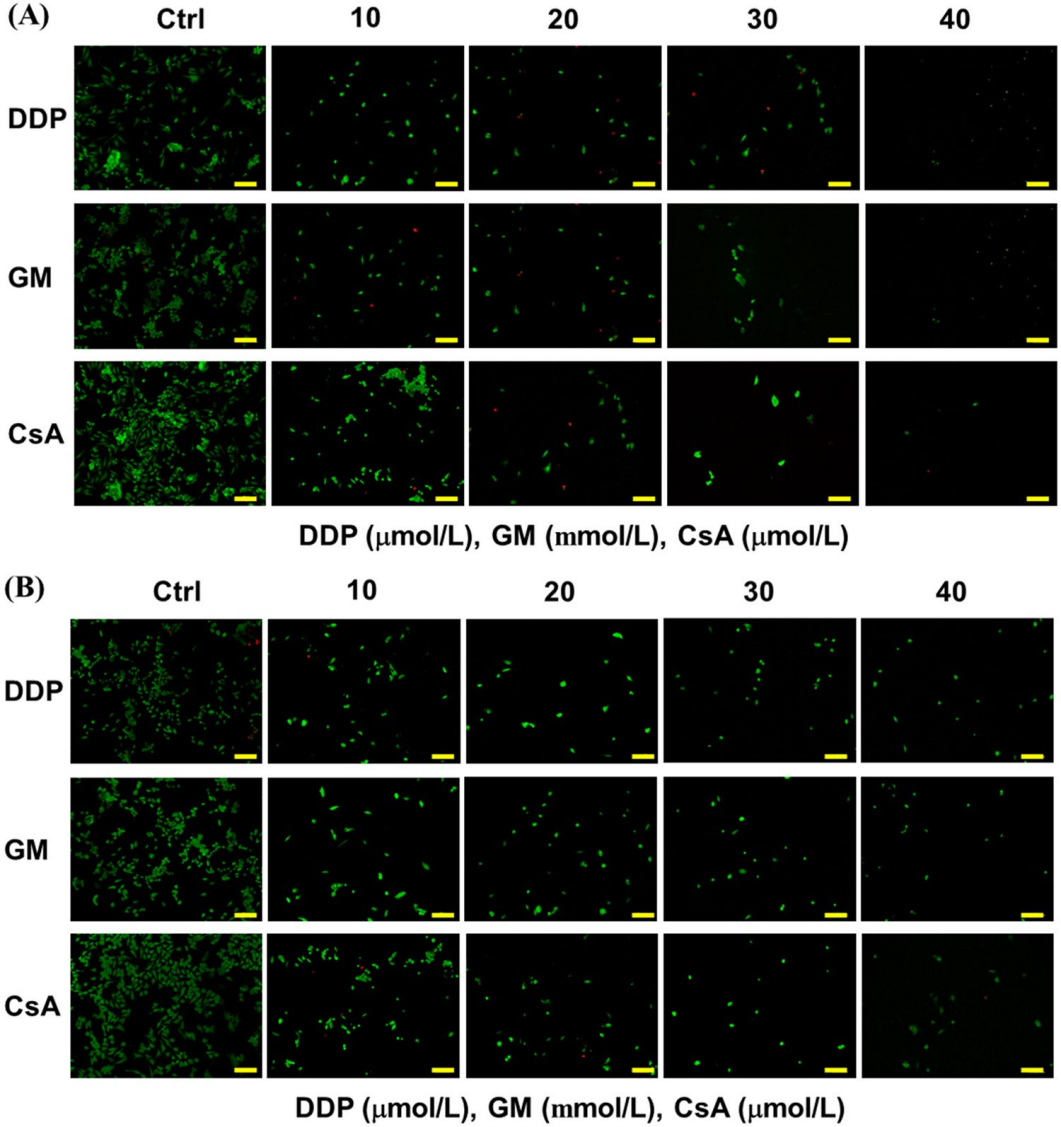
Fluorescence staining picture of RPTECs under different concentrations of DDP (μmol/L), GM (mmol/L), and CsA (μmol/L) under (A) static and (B) fluidic conditions. Scale bar = 200 μm.

Generally, cell growth and apoptosis are two reliable Indicators of drug screening in studies of drug-induced nephrotoxicity. Live/dead staining images revealed the increased cell death when the drug concentration was increased (Fig. 8). The higher drug doses would damage cells and cause nephrotoxicity. Compared with the number of cells in the static group, the continuous flow on the chip and the provision of fresh culture medium improved the cell survival rate.

**Figure 9:**
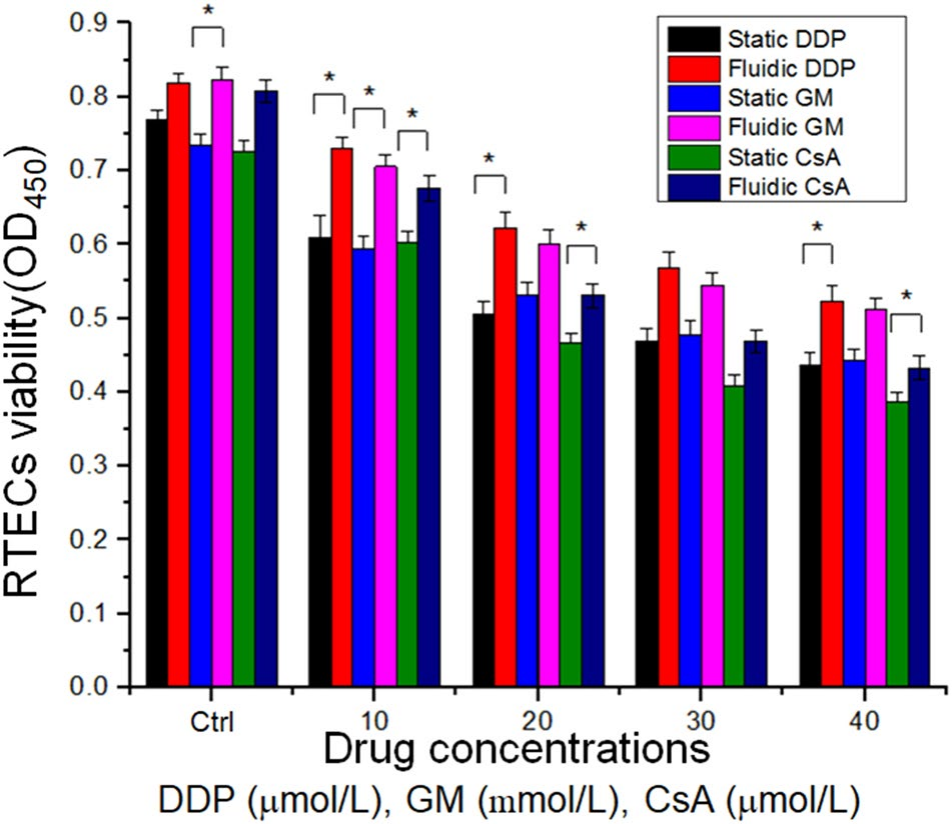
Statistical analysis. CCK-8 assay of DDP (μmol/L), GM (mmol/L), and CsA (μmol/L) under static and fluidic conditions by using a microplate reader. (*P < 0.05, **P < 0.01).

The effects of DDP, GM, and CsA on the microfluidic kidney chip were compared with those on the Petri dishes (Fig. 9). The continuous fresh liquid was poured into the chip, which made the cell get enough nutrients. The cell viability on the chip was higher than that on the dish. When the drug concentration increased, the activity of cells decreased. For example, the cell viability on the chip in the DDP group was approximately 1.55 (OD_450_). When we increased the DDP concentration to 40 μmol/L, the cells were damaged and their viability reduced to 0.81 (OD_450_). The cells treated with GM and CSA showed the same trend both on the chip and in the culture dish

### Modeling Cim to reduce the DDP-induced toxicity

**Figure 10:**
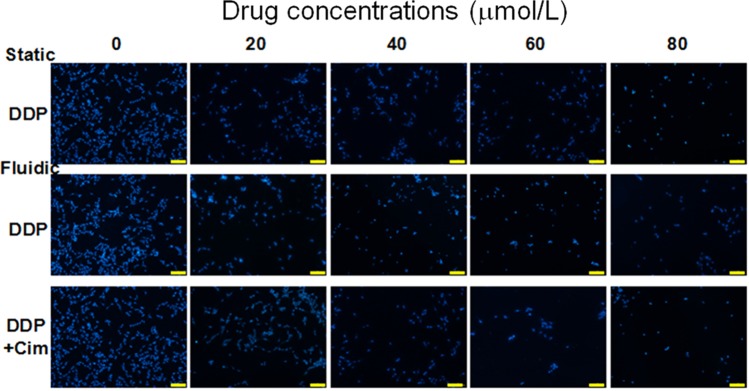
DAPI staining of RPTECs in two groups: DDP (0, 20, 40, 60, and 80 μmol/L) and DDP + Cim (0, 20, 40, 60, and 80 μmol/L). Scale bar = 200 μm.

In clinical applications, DDP can cause damage to RPTECs, while Cim can reduce the damage caused by DDP to cells. In the process of reducing toxicity, Cim plays the role of neutralizing DDP toxicity. As we expected, when DDP was added, the cell survival rate decreased. When we added Cim to the cell culture medium, Cim neutralized and reduced the toxicity of DDP, thus improving the survival rate of renal cells. In Fig. 10, We compared the cell viability of RPTECs cultured in Petri dish, microfluidic. It can be seen from the trend of the cells number that the toxicity of DDP decreased after adding Cim under the same drug concentration.

When the cells start to grow, some places look sparse. After a period of time, the cells will see denser growth under the microscope. Here, we select a representative area to show the growth trend after the drug acts on the cells. In Fig. 10, we can roughly see the growth of cells under the action of different drugs. From the analysis of CCK-8, we can obtain quantitative data on cell conditions and growth trends. The role of Cim is to neutralize the toxicity of DDP. After adding cim, the damage to the cells is reduced, and the viability of the cells is increased.

**Figure 11:**
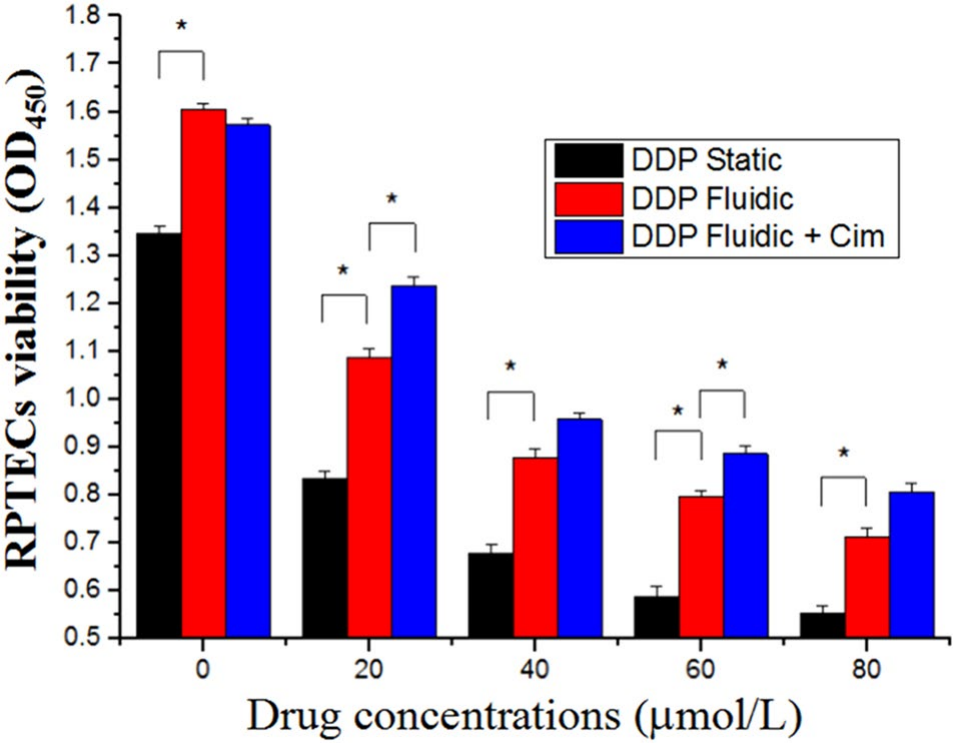
Cellular injury measured using CCK-8 assay of DDP in the presence or absence of 1 mm/L of Cim for 24 h under static or flow conditions. (*P < 0.05).

In Fig. 11, if we compare the culture of kidney cells on the Petri dish (black bar) and on-chip (red bar), we can see that the survival rate of RPTECs on-chip is higher. Fresh culture medium and flowing environment are helpful to enhance the activity of renal cells. If we compare the effect of adding Cim to the chip (red bar and blue bar), the RPTECs activity increases after adding Cim. That is to say, the toxicity of DDP was neutralized by CIM, which, as we expected, reproduced the attenuated model of renal drugs on the chip.

## Discussion

Nowadays, organ-on-chip technologies are maturing to overcome the limitations of traditional methods for drug screening. Nevertheless, these systems have limitations in the areas such as duplicate *in vivo* environment, long-term cell culture, complex operation processes, and efficiency in drug assay. Manual operations and the need of disease models are still inevitable, though the microfluidic technology provides convenient methods for artificial tissue on-chip. This study has demonstrated an integrated microfluidic system to automate part of the process, including gradient drug concentrations, long-term cell culture, and metabolite collection. More importantly, it has been demonstrated that the cimetidine intervention can decrease cisplatin-induced nephrotoxicity on kidney chip. Such a disease treatment method is a step further than measuring a drug’s response by culturing cells on a chip. All these show that the device system reported in this paper has a potential to be applied to other drug screening, including the disease model of pathogenesis and treatment.

The *In vitro* models, as developed in this study, which recapitulate critical aspects of kidney physiologic function, response to drug damage, drug resistance, and toxicity reduction will accelerate drug discovery and development. The development of a controllable kidney biomimetic microfluidic culture system provides a unique opportunity to investigate the basic kidney response. Such a system provides a flexible method for drug toxicity screening. The concept of kidney-on-chip could ultimately enable “virtual clinical trials in a chip.”

In general, the advantage of the kidney system developed in this study is not only to integrate different microfluidic designs but also to be used for other drug screening. This standardized, automatic, and low-cost method would dramatically accelerate the process of drug assays and reduce the labor cost with no human bias (as opposed to manual processes).
